# Supplementary material for: A general approach for stabilizing nanobodies for intracellular expression
Source: eLife. 2022 Nov 23;11:e68253. doi: 10.7554/eLife.68253 (PMC9683787; doi:10.7554/eLife.68253)
Supplement: Figure 2—source data 1. [file elife-68253-fig2-data1.docx]

Table 1: framework sequence variability (compared to consensus sequence) across stable and unstable nanobodies

**Unstable NBs**

extra disulfide

|  | IMGT # 11111-11111111111 |  |  |
| --- | --- | --- | --- |
|  | 11111111222222222-34444444444555555-666677777777778888888888999999999900000-11222222222 |  |  |
|  | 12345678901234567890123456-90123456789012345-678901234567890123456789012345678901234-89012345678 |  |  |
|  | **MAQVQLQESGG-GLVQAGGSLRLSCAAS-MGWFRQAPGKEREFVAA-TYYADSVKGRFTISRDNAKNTVYLQMNSLKPEDTAVYYC-WGQGTQVTVSS** | animal | CDR3 length |
| **3ZKQ** | - P - T V G W ST- T R L -R P | llama | 5 |
| **3K1K** | V -A P - R Y W G-SS E D R - | dromedary | 8 |
| **3K7U** | - T - -LF N R T - | llama | 10 |
| **3CFI** | - P - S V GL W SG- S TAP IL R -R | llama | 11 |
| **4LGP** | V T - P T G -I H WLVC- V V A L D GI - | alpaca | 11 |
| **4MQS** | - D -I Q G SC- I A S E V - | llama | 11 |
| **1ZV5** | D V - S E -I D G VF- Q S - | dromedary | 14 |
| **2P42** | V - - G - L KG D T - | dromedary | 14 |
| **3V0A** | V - P - S V EGF W SS-AWDG A T D L SN Q G - | llama | 14 |
| **4QKX** | - - Y Q L - N N A - | llama | 14 |
| **4WEU** | - - K Y - IA L D - | llama | 14 |
| **4X7C** | D V - P - Y Q L S- N G -R R | alpaca | 14 |
| **1KXV** | V - T P - S Y R G D SG- T V A QG A D D M - | dromedary | 15 |
| **4LHJ** | V - - Y L T-SN G S - | alpaca | 15 |
| **4OCL** | - P VD - A T- R - R | llama | 15 |
| **4WEM** | - E - Q GY-LN G F SN S G F - K | llama | 15 |
| **4WEN** | - P T - Y SK H- EF T D - K | llama | 15 |
| **5IV0** | V - P T T -I G SC- FMN D I - | alpaca | 15 |
| **1KXQ** | V - S S -V G - S L Q N GI - | dromedary | 16 |
| **4HEM** | V - - P T- RN NM - | llama | 16 |
| **4LAJ** | V - P -I G SC- -Y K | llama | 16 |
| **4C58** | - S G - A G C-S S R Q T AF L S I -A | llama | 17 |
| **4KML** | - P - G S-SD T M N T - | llama | 17 |
| **4KRM** | K E - S T T - SG- G D I - | llama | 17 |
| **2X6M** | G V - S - R G R- A D E I - | dromedary | 18 |
| **4W6X** | - S T - G C-S Q D F R I - | llama | 18 |
| **3K74** | - P - Y V R GL W SM- K E L TS -K | llama | 19 |
| **4GRWf** | E V - P -I G SC- ES - | llama | 19 |
| **4W6W** | - S - G C-VN Q S K L E L S - | llama | 19 |
| **4I13** | - A -I - V GE E I MN V R N - R | llama | 20 |
| **4S10** | - - S- R A K S DN N D - | llama | 20 |
| **1G6V** | V - S - G T- G Q I -R | dromedary | 21 |
| **1KXT** | VA - S - Y C LS R-AN A A D - | dromedary | 21 |
| **1RJC** | E A - S Q T - G V- A Q L L L M - | dromedary | 21 |
| **3JBC** | - S T - G G-A Q K I - | dromedary | 21 |
| **4I0C** | - S E - A G -P Q RM E M - | dromedary | 21 |
| **4LGS** | V - S - A S- L AL N - | alpaca | 22 |
| **4QGY** | V - -I G SC- P A S K M - K | llama | 22 |
| **4W6Y** | - S - A V G S- S T - | llama | 22 |
| **4HEP** | D V - P E -I G SY- V T S L - K L | llama | 24 |
| **3G9A** | D - S - A C L SN- T G D VN S R - K | dromedary | 25 |
| **1JTP** | D A - S - G - Q L E I - | dromedary | 26 |
|  |  |  |  |
|  | IMGT # 11111-11111111111 | **Stable NBs** |  |
|  | 11111111222222222-34444444444555555-666677777777778888888888999999999900000-11222222222 |  |  |
|  | 12345678901234567890123456-90123456789012345-678901234567890123456789012345678901234-89012345678 |  |  |
|  | **MAQVQLQESGG-GLVQAGGSLRLSCAAS-MGWFRQAPGKEREFVAA-TYYADSVKGRFTISRDNAKNTVYLQMNSLKPEDTAVYYC-WGQGTQVTVSS** | animal | CDR3 length |
| **2XT1** | V - - A Y A LI - V D T DD IL D M - | alpaca | 6 |
| **4X7F** | D V - P - A Y G EQ L V- D M L SN R - | alpaca | 7 |
| **4ORZ** | - - Y Q L F- D P V - S | llama | 10 |
| **4EIG** | - K - T Y L L- MTV VQ E N - | llama | 11 |
| **4CDG** | - - A Y T Q RI I- N V D M I - R | llama | 13 |
| **4LGR** | V - P H -TC Y GT Q L - ID - | alpaca | 13 |
| **4M3K** | - P - Y D G L L- T G E S - | llama | 14 |
| **3P0G** | - - Y Q L - N N A - | llama | 15 |
| **4C57** | - P S - S V RV GL W G-AH R ML S SD GL -SS | llama | 15 |
| **4WGV** | - -AN Y P MQ L T-AN R R G - | llama | 15 |
| **2BSE** | - T - LA P L V - V SG I - | llama | 16 |
| **4GRWh** | E V - -V -P D R L - | llama | 16 |
| **4IOS** | V - D V -I - GR M CAA A L - | llama | 16 |
| **4NBX** | V - A - A P - - | llama | 16 |
| **4NC2** | V - - -PN S Q - | llama | 16 |
| **4P2C** | - V - Y Q S- N R - | llama | 16 |
| **4QO1** | - - R L Q- E D T E N NAD GI F - | llama | 16 |
| **1OP9** | - S S -S G - Q M - E | dromedary | 17 |
| **3EBA** | V - S S -S GL W - Q M - | dromedary | 17 |
| **4AQ1** | - - G- GA M G A - | llama | 17 |
| **4GFT** | - T P K S A- Q G-Q M A V A - | llama | 17 |
| **4NBZ** | K E - -VA A V- V N R F - | llama | 17 |
| **1ZVH** | D V - S -L G -P V L E L - | dromedary | 18 |
| **4GRWe** | E V - P -IA G SG-A S R - K L | llama | 18 |
| **1ZVY** | D V - S - T A G - T Q K MA R D V S I T - | dromedary | 20 |
| **3RJQ** | - T - - D A K A - | llama | 20 |
| **4DK3** | - - - T E - | llama | 20 |
| **4N1H** | - A K - - K A D W R - | llama | 20 |
| **4TVS** | V - -V L T- H - | alpaca | 20 |
| **4EIZ** | - T - T - L M - | llama | 21 |
| **4FHB** | - E - D V- A I - | llama | 22 |
| **4LHQ** | V T - T T S - - R V HL L A - | alpaca | 22 |
| **4KRO** | - P - Q - T T - | llama | 23 |

*all nanobodies contain a conserved set of cysteines that normally forms a disulfide bond through the hydrophobic core of the nanobody (23Cys and 104Cys). Highlighted in yellow, “extra disulfide bond” refers to nanobodies with an additional pair of cysteines, one of which is always located in CDR3, that normally forms a disulfide bond that impacts CDR3 conformation.
