# Supplementary material for: A general approach for stabilizing nanobodies for intracellular expression
Source: eLife. 2022 Nov 23;11:e68253. doi: 10.7554/eLife.68253 (PMC9683787; doi:10.7554/eLife.68253)
Supplement: Figure 4—source data 1. [file elife-68253-fig4-data1.docx]

Table 2A: pre-mutagenesis framework sequence variability across unstable nanobodies

unstable

partially stable

|  | IMGT # 1111111111111111 |
| --- | --- |
|  | 11111111122222223444444444455555566667777777777888888888899999999990000011222222222 |
|  | 12345678912345678901234569012345678901234567890123456789012345678901234567890123489012345678 |
| **NB#** | **MAQVQLQESGGGLVQAGGSLRLSCAASMGWFRQAPGKEREFVAATYYADSVKGRFTISRDNAKNTVYLQMNSLKPEDTAVYYCWGQGTQVTVSS** |
| 1 | **1G6V** V S G T G Q I R |
| 2 | **1JTP** D A S G Q L E I |
| 3 | **1KXQ** V S S V G S L Q N GI |
| 4 | **1KXT** VA S Y C LS RAN A A D |
| 5 | **1KXV** V T P S Y R G D SG T V A QG A D D M |
| 6 | **1RJC** E A S Q T G V A Q L L L M |
| 7 | **1ZV5** D V S E I D G VF Q S |
| 8 | **2P42** V G L KG D T |
| 9 | **2X6M** G V S R G R A D E I |
| 10 | **3CFI** P S V GL W SG S TAP IL R R |
| 11 | **3G9A** D S A C L SN T G D VN S R K |
| 12 | **3JBC** S T G GA Q K I |
| 13 | **3K1K** V A P R Y W GSS E D R |
| 14 | **3K74** P Y V R GL W SM K E L TS K |
| 15 | **3K7U** T LF N R T |
| 16 | **3V0A** V P S V EGF W SSAWDG A T D L SN Q G |
| 17 | **3ZKQ** P T V G W ST T R L R P |
| 18 | **4C58** S G A G CS S R Q T AF L S I A |
| 19 | **4GRWf** E V P I G SC ES |
| 20 | **4HEM** V P T RN NM |
| 21 | **4HEP** D V P E I G SY V T S L K L |
| 22 | **4I0C** S E A G P Q RM E M |
| 23 | **4I13** A I V GE E I MN V R N R |
| 24 | **4KML** P G SSD T M N T |
| 25 | **4KRM** K E S T T SG G D I |
| 26 | **4LAJ** V P I G SC Y K |
| 27 | **4LGP** V T P T G I H WLVC V V A L D GI |
| 28 | **4LGS** V S A S L AL N |
| 29 | **4LHJ** V Y L TSN G S |
| 30 | **4MQS** D I Q G SC I A S E V |
| 31 | **4OCL** P VD A T R R |
| 32 | **4QGY** V I G SC P A S K M K |
| 33 | **4QKX** Y Q L N N A |
| 34 | **4S10** S R A K S DN N D |
| 35 | **4W6W** S G CVN Q S K L E L S |
| 36 | **4W6X** S T G CS Q D F R I |
| 37 | **4W6Y** S A V G S S T |
| 38 | **4WEM** E Q GYLN G F SN S G F K |
| 39 | **4WEN** P T Y SK H EF T D K |
| 40 | **4WEU** K Y IA L D |
| 41 | **4X7C** D V P Y Q L S N G R R |
| 42 | **5IVN** V P T T I G SC FMN D I |
