# Supplementary material for: A general approach for stabilizing nanobodies for intracellular expression
Source: eLife. 2022 Nov 23;11:e68253. doi: 10.7554/eLife.68253 (PMC9683787; doi:10.7554/eLife.68253)
Supplement: Figure 5—source data 1. [file elife-68253-fig5-data1.docx]

Table 2B: sequence and stability variability across nanobodies following stage 1 mutagenesis

unstable

partially stable

stable

|  | IMGT# 1111111111111111 |
| --- | --- |
|  | 11111111122222223444444444455555566667777777777888888888899999999990000011222222222 |
|  | 12345678912345678901234569012345678901234567890123456789012345678901234567890123489012345678 |
| **MUT#** | **MAQVQLQESGGGLVQAGGSLRLSCAASMGWFRQAPGKEREFVAATYYADSVKGRFTISRDNAKNTVYLQMNSLKPEDTAVYYCWGQGTQVTVSS** |
| 7 | **1KXQ** V V ***G*** I |
| 6 | **1KXT** V Y C L RAN |
| 11 | **1KXV** V S Y G G T V M |
| 4 | **2X6M** V R ***G*** R A I |
| 8 | **3CFI** P S G W G S T |
| 9 | **3G9A** A C L N T R |
| 4 | **3K1K** V P R Y W GSS E |
| 9 | **3K74** P Y G W M K |
| 13 | **3V0A** V P S G W SAW G |
| 6 | **3ZKQ** P T G W T T |
| 4 | **4GRFf** V P I ***G*** C |
| 8 | **4HEP** V P E I ***G*** Y |
| 9 | **4I13** I V G N |
| 2 | **4KML** P G SSD T T |
| 7 | **4KRM** G G I |
| 3 | **4LGS** V A S L A |
| 2 | **4LHJ** V Y L TSN |
| 7 | **4MQS** I ***G*** C I |
| 4 | **4OCL** V A T |
| 5 | **4QGY** V I ***G*** C P M |
| 2 | **4QKX** Y Q L N |
| 7 | **4S10** S R |
| 8 | **4WEM** E Q YLN G |
| 5 | **4WEN** P T Y K H E |
| 4 | **4WEU** K Y |
| 4 | **4X7C** V P Y Q L S N |
| 3 | **1G6V** V ***G*** T G I |
| 9 | **1RJC** ***G*** V A M |
| 5 | **1ZV5** V I D ***G*** F S |
| 4 | **3J6A** ***G*** GA I |
| 3 | **4I0C** E A ***G*** P R M |
| 6 | **4W6W** ***G*** CVN ***E*** L |
| 5 | **1JTP** ***G*** ***L*** I |
| 3 | **2P42** V ***G*** L T |
| 4 | **3K7U** LF |
| 9 | **4C58** G ***G*** CS S ***L*** I |
| 4 | **4HEM** V T R |
| 3 | **4LAJ** V P I ***G*** C |
| 10 | **4LGP** V P I W C V V I |
| 4 | **4W6X** T ***G*** CS ***R*** I |
| 2 | **4W6Y** A V ***G*** S T |
| 6 | **5IVN** V P T I ***G*** C I |

*52Gly and 90X are bolded and italicized. The number of mutations made to each nanobody is reported in the left-most column
