# Supplementary material for: A general approach for stabilizing nanobodies for intracellular expression
Source: eLife. 2022 Nov 23;11:e68253. doi: 10.7554/eLife.68253 (PMC9683787; doi:10.7554/eLife.68253)
Supplement: Figure 6—source data 1. [file elife-68253-fig6-data1.docx]

Table 2C: sequence and stability variability across nanobodies following stage 2 mutagenesis (+G52F +X90Q)

unstable

partially stable

stable

|  | IMGT# 1111111111111111 |
| --- | --- |
|  | 11111111122222223444444444455555566667777777777888888888899999999990000011222222222 |
|  | 12345678912345678901234569012345678901234567890123456789012345678901234567890123489012345678 |
| **MUT#** | **MAQVQLQESGGGLVQAGGSLRLSCAASMGWFRQAPGKEREFVAATYYADSVKGRFTISRDNAKNTVYLQMNSLKPEDTAVYYCWGQGTQVTVSS** |
| 4 | **1G6V** V T G I |
| 7 | **1JTP** I |
| 10 | **1RJC** V A M |
| 6 | **1ZV5** V I D F S |
| 5 | **3J6A** GA I |
| 4 | **4I0C** E A P R M |
| 4 | **4LAJ** V P I C |
| 8 | **4W6W** CVN L |
| 6 | **4W6X** T CS I |
| 3 | **4W6Y** A V S T |
| 7 | **5IVN** V P T I C I |
| 4 | **2P42** V L T |
| 4 | **3K7U** LF |
| 11 | **4C58** G CS S I |
| 4 | **4HEM** V T R |
| 10 | **4LGP** V P I W C V V I |
