# Supplementary material for: A general approach for stabilizing nanobodies for intracellular expression
Source: eLife. 2022 Nov 23;11:e68253. doi: 10.7554/eLife.68253 (PMC9683787; doi:10.7554/eLife.68253)
Supplement: Supplementary file 1. [file elife-68253-supp1.docx]

Supplemental Table 1: list of nanobodies and their associated antigens

| **NB (PDB)** | **Sequence** | **Target** |
| --- | --- | --- |
| **4OCL** | MAQVQLQESGGGLVPAGGSLRLSCVDSGRTFSSTVMAWFRQAPGKEREFVATIRWSGGNTYYADSVKGRFTISRDNARNTVYLQMNSLKPEDTAVYYCAGGTYYGTLSYKYDFWGRGTQVTVS | 26S proteasome regulatory subunit RPN8 |
| **1KXT** | MAQVQLVASGGGSVQAGGSLRLSCAASGYTFSSYPMGWYRQAPGKECELSARIFSDGSANYADSVKGRFTISRDNAANTAYLQMDSLKPEDTAVYYCAAGPGSGKLVVAGRTCYGPNYWGQGTQVTVS | alpha amylase, pancreatic |
| **1KXV** | MAQVQLVESGGGTVPAGGSLRLSCAASGNTLCTYDMSWYRRAPGKGRDFVSGIDNDGTTTYVDSVAGRFTISQGNAKNTAYLQMDSLKPDDTAMYYCKPSLRYGLPGCPIIPWGQGTQVTVS | alpha amylase, pancreatic |
| **1KXQ** | MAQVQLVESGGGSVQAGGSLSLSCAASTYTDTVGWFRQAPGKEREGVAAIYRRTGYTYSADSVKGRFTLSQDNNKNTVYLQMNSLKPEDTGIYYCATGNSVRLASWEGYFYWGQGTQVTVS | alpha amylase, pancreatic |
| **2X6M** | MAQGQLVESGGGSVQAGGSLRLSCAASGIDSSSYCMGWFRQRPGKEREGVARINGLGGVKTAYADSVKDRFTISRDNAENTVYLQMNSLKPEDTAIYYCAAKFSPGYCGGSWSNFGYWGQGTQVTVS | alpha-synuclein |
| **3ZKQ** | MAQVQLQESGGGLVQPGGSLRLSCAASGFTFSSAIMTWVRQAPGKGREWVSTIGSDGSITTYADSVKGRFTISRDNARNTLYLQMNSLKPEDTAVYYCTSAGRRGPGTQVTVS | BACE2 |
| **4QKX** | MAQVQLQESGGGLVQAGGSLRLSCAASGSIFALNIMGWYRQAPGKQRELVAAIHSGGTTNYANSVKGRFTISRDNAANTVYLQMNSLKPEDTAVYYCNVKDFGAIIYDYDYWGQGTQVTVS | beta2 adrenoceptor |
| **3P0G** | MAQVQLQESGGGLVQAGGSLRLSCAASGSIFSINTMGWYRQAPGKQRELVAAIHSGGSTNYANSVKGRFTISRDNAANTVYLQMNSLKPEDTAVYYCNVKDYGAVLYEYDYWGQGTQVTVS | beta2 adrenoceptor |
| **5IVN** | MAQVQLVESGGGLVQPGGSLTLSCTASGFTLDHYDIGWFRQAPGKEREGVSCINNSDDDTYYADSVKGRFTIFMNNAKDTVYLQMNSLKPEDTAIYYCAEARGCKRGRYEYDFWGQGTQVTVS | beta-catenin |
| **4M3K** | MAQVQLQESGGGLVQPGGSLRLSCAASGSISSITTMGWYRQDGRELVALINSVGDTTYAGSVKGRFTISRDNAKNTVYLEMSSLKPEDTAVYYCNAFMSTNSGRTGSFWGQGTQVTVS | Blap beta-lactamase (Bacillus licheniformis) |
| **4N1H** | MAQVQLQESGGGLVQAGASLKLSCAASGRTFSSYAMGWFRQAPGKEREFVAAISRSGGDTKYADSVKGRFAISRDNDKNTVWLRMNSLKPEDTAVYYCAATTYASLSDTYIGEHIYDDWGQGTQVTVS | Blap beta-lactamase (Bacillus licheniformis) |
| **4CDG** | MAQVQLQESGGGLVQAGGSLRLSCAASGIWFSINNMAWYRQTPGKQRERIAIITSAGTTNYVDSVKGRFTISRDDAKNTMYLQMNSLIPEDTAVYYCNLVADYDMGFQSFWGRGTQVTVS | blm (helicase) |
| **3V0A** | MAQVQLVESGGGLVQPGGSLRLSCAASGFTLGSRYMSWVRQAPGEGFEWVSSIEPSGTAWDGDSAKGRFTTSRDDAKNTLYLQMSNLQPEDTGVYYCATGYRTDTRIPGGSWGQGTQVTVS | botulinum neurotoxin |
| **1G6V** | MAQVQLVESGGGSVQAGGSLRLSCAASGYTVSTYCMGWFRQAPGKEREGVATILGGSTYYGDSVKGRFTISQDNAKNTVYLQMNSLKPEDTAIYYCAGSTVASTGWCSRLRPYDYHYRGQGTQVTVS | bovine carbonic anhydrase |
| **3K74** | MAQVQLQESGGGLVQPGGSLRLSCAASGFTFNNYWMYWVRRAPGKGLEWVSMINPGGIITKYAESVKGRFTISRDNAKNTLYLQMNSLTSEDTAVYYCAKDWATGLAKKGQGTQVTVS | DHFR (*E. coli*) |
| **4EIG** | MAQVQLQESGGGLVQAGGSLRLSCKASGIIFSVYKMTWYRQAPGKERELVALITTNNNTMTVDSVKGRFTISRDNVQNTVYLEMNNLKPEDTAVYYCNANRGLAGPAYWGQGTQVTVS | DHFR (*E. coli*) |
| **4EIZ** | MAQVQLQESGGGLVQAGGSLRLSCTASGRTFSSYAMGWFRQTPGKEREFVAAITWGGSTTLYADSVKGRFTMSRDNAKNTVYLQMNSLKPEDTAVYYCAADGSQYRSTYSFRDKPDYGSWGQGTQVTVS | DHFR (*E. coli*) |
| **4FHB** | MAQVQLQESGGGLVQAGGSLRLSCEASGRTFSSYAMGWFRQAPERDFVAVISWSGSNTYYADSAKGRFTISRDNAKNTVYLQMNSLKPEDTAIYYCAAPGRPHGSSWSLNKKGQGYDYWGQGTQVTVS | DHFR (*E. coli*) |
| **4I13** | MAQVQLQESGGGLVQAGASLRLSCAASERLTVDYAIGWFRQAPGKEREFVAAISWGGGLTVYGESVEGRFTISRDIAKNTMNLQMNVLRPEDTANYYCAASRISYRVWNTIPYNKLTLWGRGTQVTVS | DHFR (*E. coli*) |
| **4WGV** | MAQVQLQESGGGLVQAGGSLRLSCAASRSIFSIDTANWYRQPPGMQRELVATITRDGNANYADSVKGRFTISRDRARNTVYLQMNSLKPEDTGVYYCNAAIRTTVRTSAQEYWGQGTQVTVS | Divalent metal cation transporter MntH |
| **4KRM** | MAQVKLEESGGGSVQTGGSLRLTCAASGRTSRSYGMGWFRQAPGKEREFVSGISWRGDSTGYADSVKGRFTISRDNAKNTVDLQMNSLKPEDTAIYYCAAAAGSAWYGTLYEYDYWGQGTQVTVS | EGFR |
| **4KRO** | MAQVQLQESGGGLVQPGGSLRLSCAASGRTFSSYAMGWFRQAPGKQREFVAAIRWSGGYTYYTDSVKGRFTISRDNAKTTVYLQMNSLKPEDTAVYYCAATYLSSDYSRYALPQRPLDYDYWGQGTQVTVS | EGFR |
| **3CFI** | MAQVQLQESGGGLVQPGGSLRLSCAASGFAFSGYAMSWVRQAPGKGLEWVSGINRDGSTSYTAPVKGRFTISRDNAKNILYLQMNSLRPEDTAVYYCAKWLGGRDWYDRGQGTQVTVS | EpsI:EpsJ pseudopilin heterodimer |
| **4WEM** | MAQVQLQESGGGLVQAGGSLRLSCEASGNVDRIDAMGWFRQAPGKQREFVGYISEGGILNYGDFVKGRFTISRDNAKNTVYLQMSNLKSEDTGVYFCAASHWGTLLIKGIEHWGKGTQVTVS | F4 fimbrial adhesin FaeG |
| **4WEN** | MAQVQLQESGGGLVQPGGSLRLSCTASGSISSINAMGWYRQAPGSKREFVAHITNTGVTEFADSVKGRFTISRDNAKTTVDLQMNSLKPEDTAVYYCAATDWGTLLIKGIDHWGKGTQVTVS | F4 fimbrial adhesin FaeG |
| **4WEU** | MAQVQLQESGGGLVQAGGSLRLSCAASGLTFDTYAMGWFRQAPGKKREYVAAISWTGISTYYADIAKGRFTISRDNAKNTLYLQMDSLKPEDTAVYYCAAQKSLNVPAPWDYWGQGTQVTVS | F4 fimbrial adhesin FaeG |
| **4C57** | MAQVQLQESGGGLVQPGGSLRLSCSASGFKFNDSYMSWVRRVPGKGLEWVAGIWEDSSAAHYRDSVKGRFTISRDNAKNMLYLQMSSLKSDDTGLYYCVRRGYSGDYRPINNPSSQGTQVTVS | GAK kinase |
| **4C58** | MAQVQLQESGGGSVQAGGSLRLSCGASEYTSRMGWFRQAPGAEREGVACIHRQSNLSYYSDSVRGRFTISQDNAKTTAFLLMSSLKPEDTAIYYCATTTDCAAFVERATAITAGQGTQVTVS | GAK kinase |
| **4S10** | MAQVQLQESGGGLVQAGGSLRLSCAASGRTFSSFVMGWFRQAPGKEREFVASISRSGSVTRYADSAKGRFTISKDNAKNTVSLQMDNLNPDDTAVYYCAADLHRPYGPGSQRTDDYDTWGQGTQVTVS | gelsolin |
| **3G9A** | MAQVQLQESGGGSVQAGGSLRLSCAASGDTFSSYSMAWFRQAPGKECELVSNILRDGTTTYAGSVKGRFTISRDDAKNTVYLQMVNLKSEDTARYYCAADSGTQLGYVGAVGLSCLDYVMDYWGKGTQVTVS | GFP |
| **3K1K** | MAQVQLVESGGALVQPGGSLRLSCAASGFPVNRYSMRWYRQAPGKEREWVAGMSSAGDRSSYEDSVKGRFTISRDDARNTVYLQMNSLKPEDTAVYYCNVNVGFEYWGQGTQVTVS | GFP |
| **3RJQ** | MAQVQLQESGGGLVQAGGSLRLSCTASGRISSSYDMGWFRQAPGKEREFVAAISWSGGTTDYADSVKGRFAISKDNAKNAVYLQMNSLKPEDTAVYYCAAKWRPLRYSDYPSNSDYYDWGQGTQVTVS | HIV C186 gp120 |
| **2XT1** | MAQVQLVESGGGLVQAGGSLRLSCAASGSFFMSNVMAWYRQAPGKARELIAAIRGGDMSTVYDDSVKGRFTITRDDDKNILYLQMNDLKPEDTAMYYCKASGSSWGQGTQVTVS | HIV Gag p24 |
| **4ORZ** | MAQVQLVESGGGLVQAGGSLRLFCAASGFTFGTSNMAWLRQAPGKRREWVALITISGYTDYADSVKDRFTISRDNAKNTVSLQMNSLKPEDTAIYFCARRVGSEYDLWGQGTQVTVS | HIV-1 Nef |
| **4LAJ** | MAEVQLVESGGGLVQPGGSLRLSCAASGFTLDYYSIGWFRQAPGKEREGVSCISDSDGRTYYADSVKGRFTISRDNAKNTVYLQMNSLKPEDTAVYYCATDCTVDPSLLYVMYYGKGTQVTVS | HIV-1 YU2 envelope gp120 glycoprotein |
| **4MQS** | MAQVQLQESGGGLVQAGDSLRLSCAASGFDFDNFDDYAIGWFRQAPGQEREGVSCIDPSDGSTIYADSAKGRFTISSDNAENTVYLQMNSLKPEDTAVYVCSAWTLFHSDEYWGQGTQVTVS | M2 muscarinic acetylcholine receptor |
| **4KML** | MAQVQLQESGGGLVQPGGSLRLSCAASGRTFSSYNMGWFRQAPGKGREFVASITSSGDKSDYTDSVKGRFTISRDNAKNTMYLQMNNLKPEDTATYYCARGLGIYIIRARGGYDHWGQGTQVTVS | human prion protein |
| **4GRWE** | MAEVQLVESGGGLVQPGGSLRLSCAASGFTLDDYAIAWFRQAPGKEREGVSGIDSGDGSAYYADSVKGRFTISSDNAKNTVYLQMNSLRPEDTAVYYCARVRTGWGLNAPDYAMDYWGKGTLVTVS | Interleukin 23 |
| **4GRWF** | MAEVQLVESGGGLVQPGGSLRLSCAASGFTLDYLAIGWFRQAPGKEREGVSCVSSSGQYTYYADSVKGRFTISRDNAESTVYLQMNSLKPEDTAVYYCATDPECYRVRGYYNGEYDYWGQGTQVTVS | Interleukin 23 |
| **4GRWH** | MAEVQLVESGGGLVQAGGSLRLSCAASGRTFSWSAVGWFRQAPGKEREFVAAIRWSGGSPYYADSVKDRFTISRDNAKNTVYLQMNSLRPEDTAVYLCGETSLFPTSRGSHYDTWGQGTQVTVS | Interleukin 23 |
| **4TVS** | MAQVQLVESGGGLVQAGGSLRLSCAASGRTLSSYAVGWFRQAPGLEREFVATISRSGGSTHYADSVKGRFTISRDNAKNTVYLQMNSLKPEDTAVYYCAATFTPDGSWYYTRGSSYDYWGQGTQVTVS | LAP1 |
| **4W6W** | MAQVQLQESGGGSVQAGGSLRLSCAASGYTSGRDSMGWFRQAPGKEREGVACIDTSGIVNYADSVKGRFTISQDSAKKTLYLEMNSLKPEDTALYSCATGPFVYGRGCLGQAFYSYWGQGTQVTVS | lectin domain of F18 fimbrial adhesin FedF |
| **4W6X** | MAQVQLQESGGGSVQAGGSLRLSCTASGYTYRKYCMGWFRQAPGKEREGVACINSGGGTSYYADSVKGRFTISQDNAKDTVFLRMNSLKPEDTAIYYCALSSNSVCPPGHVAWYNDWGQGTQVTVS | lectin domain of F18 fimbrial adhesin FedF |
| **4W6Y** | MAQVQLQESGGGSVQAGGSLRLSCAASGYTYSSNCMAWFRQVPGKEREGVASINTRGGITYYADSVKGRFTISRDNAKNTVSLQMNSLKPEDTATYYCAAVREATYSDNRCSVRSYTYDYWGQGTQVTVS | lectin domain of F18 fimbrial adhesin FedF |
| **1JTP** | MADVQLQASGGGSVQAGGSLRLSCAASGYTIGPYCMGWFRQAPGKEREGVAAINMGGGITYYADSVKGRFTISQDNAKNTVYLLMNSLEPEDTAIYYCAADSTIYASYYECGHGLSTGGYGYDSWGQGTQVTVS | lysozyme (hen) |
| **1RJC** | MAQVQLQASGGGSVQAGQSLRLSCATSGATSSSNCMGWFRQAPGKEREGVAVIDTGRGNTAYADSVQGRLTISLDNAKNTLYLQMNSLKPEDTAMYYCAADTSTWYRGYCGTNPNYFSYWGQGTQVTVS | lysozyme (hen) |
| **1ZV5** | MADVQLVESGGGSVQAGESLRLSCAASGVTYKNYCIGWFRQAPGKDREGVVFINSDGGITYYADSVKGRFTISQDNAKNTVYLQMNSLKPEDTASYYCAAGYRNYGQCATRYWGQGTQVTVS | lysozyme (hen) |
| **1ZVH** | MADVQLVESGGGSVQAGGSLRLSCAASGYIASINYLGWFRQAPGKEREGVAAVSPAGGTPYYADSVKGRFTVSLDNAENTVYLQMNSLKPEDTALYYCAAARQGWYIPLNSYGYNYWGQGTQVTVS | lysozyme (hen) |
| **1ZVY** | MADVQLVESGGGSVQAGGSLRLSCAASGSTDSIEYMTWFRQAPGKAREGVAALYTHTGNTYYTDSVKGRFTISQDKAKNMAYLRMDSVKSEDTAIYTCGATRKYVPVRFALDQSSYDYWGQGTQVTVS | lysozyme (hen) |
| **1OP9** | MAQVQLQESGGGSVQAGGSLRLSCSASGYTYISGWFRQAPGKEREGVAAIRSSDGTTYYADSVKGRFTISQDNAKNTVYLQMNSLKPEDTAMYYCAATEVAGWPLDIGIYDYWGQGTEVTVS | lysozyme (human) |
| **3EBA** | MAQVQLVESGGGSVQAGGSLRLSCSASGYTYISGWFRQAPGKGLEWVAAIRSSDGTTYYADSVKGRFTISQDNAKNTVYLQMNSLKPEDTAMYYCAATEVAGWPLDIGIYDYWGQGTQVTVS | lysozyme (human) |
| **4I0C** | MAQVQLQESGGGSVQAGGSLRLSCEASGLSTTVMAWFRQAPGKEREGVAAIYTGDGFPYYADSVKGRFTISQDNAKNRMYLQMNSLEPEDTAMYYCAAKTGAFSYGSLWWMSRAYNHWGQGTQVTVS | lysozyme (human) |
| **4GFT** | MAEVQLQESGGGTVQPGGSLKLSCSAAPERAFSNYAMGWFRQAPGQEREFVAGITGSGRSQYYADSVKGRFTISRDNAMNAVYLQMNSVKAEDTAVYYCAARVVPVFSDSTKGYVYWGQGTQVTVS | malaria invasion machinery protein |
| **3K7U** | MAQVQLQESGGGLVQAGGSLTLSCAASGRTFSNNAMGWFRQAPGKEREFVAAISWTGGLLFYADSVNGRFTISRDNAKRTVTLQMNSLKPEDTAVYYCAARPQGDYVTAHYDYWGQGTQVTVS | MP18 RNA editing complex protein |
| **4DK3** | MAQVQLQESGGGLVQAGGSLRLSCAASGRTSSLYSMGWFRQAPGKEREFVAAISRNGANTYYTDSVKGRFTISRDNAKNTVELQMNSLKPEDTAVYYCAADRFPTMEVVTIMTNEYDYWGQGTQVTVS | MP18 RNA editing complex protein |
| **4X7F** | MADVQLVESGGGLVQPGGSLRLSCAASESILSFNHMAWYRQGPGEQRELVAVITREGSTDYADSVKGRFTISRDNAKNMVYLLMSNLRPEDTAVYYCNRGISNPWGQGTQVTVS | norovirus GII.10 P |
| **4X7C** | MADVQLVESGGGLVQPGGSLRLSCAASGSIFSIYAMGWYRQAPGKQRELVASISSGGGTNYADSVKGRFTISGDNAKNTVYLQMNSLKPEDTAVYYCKREDYSAYAPPSGRGRGTQVTVS | Norwalk virus VP1 |
| **2BSE** | MAQVQLQESGGGLVQAGGSLRLSCTASRRTGSNWCMGWFRQLAGKEPELVVALNFDYDMTYYADSVKGRFTVSRDSGKNTVYLQMNSLKPEDTAIYYCAARSGGFSSNRELYDGWGQGTQVTVS | p2 receptor binding protein (Lactococcal Bacteriophage) |
| **4QO1** | MAQVQLQESGGGLVQAGGSLRLSCAASERTFSTYAMGWFRQAPGREREFLAQINWSGTTTYYAESVKDRTTISRDNAKNTVYLEMNNLNADDTGIYFCAAHPQRGWGSTLGWTYWGQGTQVTVS | P53 DBD |
| **4IOS** | MAQVQLVESGGGLVQAGDSLRLSCAVSSNVIGWFRQAPGKEREFVAAISWSTGSTYYGRSMKGRCAASRDNTVALQLNSLKPEDTAVYYCAATLDWGKTLSDEYDYWGQGTQVTVS | phage TP901-1 RBP |
| **3J6A** | MAQVQLQESGGGSVQTGGSLRLSCAASEYTQSSACMGWFRQAPGKEREGVAGISRFFGTAYYADSVKGRFTISQDKAKNTVYLQMNSLKPEDTAIYYCAAGQGCLTTIQALGGAYGYNAWGQGTQVTVS | poliovirus capsid (hetero-protein) |
| **4HEM** | MAQVQLVESGGGLVQAGGSLRLSCAASESTFSNYAMGWFRQAPGPEREFVATISQTGSHTYYRNSVKGRFTISRDNAKNTVYLQMNNMKPEDTAVYYCAAGDNYYYTRTYEYDYWGQGTQVTVS | RBP from lactococcal phage TP901-1 |
| **4HEP** | MADVQLVESGGGLVQPGGSLRLSCEASGFSFDDYAIGWFRQAPGKEREGVSYISMSDGRTYVADSVTGRFTISSDNAKNTVYLQMNSLKLEDTAVYYCAAGRFVTFGSAWSFVGGGPYGIDYWGKGTLVTVS | RBP from lactococcal phage TP901-1 |
| **4LGP** | MAQVQLVETGGGLVQPGGSLTLSCAGSGGTLEHYAIGWFRQAPGKEHEWLVCNRGEYGSTVYVDSVKGRFTASRDNAKNTVYLQLNSLKPDDTGIYYCVSGCYSWRGPWGQGTQVTVS | ricin A chain |
| **4LGR** | MAQVQLVESGGGLVQPGGSLRLHCAASGSIASIYRTCWYRQGTGKQRELVAAITSGGNTYYADSVKGRFTISRDNAKNTIDLQMNSLKPEDTAVYYCNADEAGIGGFNDYWGQGTQVTVS | ricin A chain |
| **4LGS** | MAQVQLVESGGGLVQAGGSLSLSCAASGGDFSRNAMAWFRQAPGKEREFVASINWTGSGTYYLDSVKGRFTISRDNAKNALYLQMNNLKPEDTAVYYCARSTVFAEITGLAGYQSGSYDYWGQGTQVTVS | ricin A chain |
| **4LHJ** | MAQVQLVESGGGLVQAGGSLRLSCAASGSIVNFETMGWYRQAPGKERELVATITNEGSSNYADSVKGRFTISGDNAKNTVSLQMNSLKPEDTAVYYCSATFGSRWPYAHSDHWGQGTQVTVS | ricin A chain |
| **4LHQ** | MAQVQLVETGGGTVQTGGSLRLSCSASGGSFSRNAMGWFRQAPGKEREFVAAINWSASSTYYRDSVKGRFTVSRDNAKNTVYLHLNSLKLEDTAAYYCAGSSVYAEMPYADSVKATSYNYWGQGTQVTVS | ricin A chain |
| **2P42** | MAQVQLVESGGGLVQAGGSLRLSCAASGYAYTYIYMGWFRQAPGKEREGVAAMDSGGGGTLYADSVKGRFTISRDKGKNTVYLQMDSLKPEDTATYYCAAGGYELRDRTYGQWGQGTQVTVS | RNASE A |
| **4AQ1** | MAQVQLQESGGGLVQAGGSLRLSCAASGRTSSAYAMGWFRQAPGKEREFVAGISSKGGSTYYGASMKGRFTISRDNAKNTVYLQMNGLAPEDTAVYYCAASDKYNFDTSHAGYGYWGQGTQVTVS | sbsB (Geobacillus stearothermophilus) |
| **4P2C** | MAQVQLQESGGGLVQAGGSLRLSCAVSGSIFRLSTMGWYRQAPGKQREFVASITSYGDTNYRDSVKGRFTISRDNAKNTVYLQMNSLKPEDTAVYYCNANIEAGTYYGPGRDYWGQGTQVTVS | Shiga toxin 2e |
| **4NBX** | MAQVQLVESGGGLAQAGGSLRLSCAASGRTFSMDPMAWFRQPPGKEREFVAAGSSTGRTTYYADSVKGRFTISRDNAKNTVYLQMNSLKPEDTAVYYCAAAPYGANWYRDEYAYWGQGTQVTVS | TcdA-A1 |
| **4NBZ** | MAQVKLEESGGGLVQAGGSLRLSCAASERTFSRYPVAWFRQAPGAEREFVAVISSTGTSTYYADSVKGRFTISRDNAKVTVYLQMNNLKREDTAVYFCAVNSQRTRLQDPNEYDYWGQGTQVTVS | TcdA-A1 |
| **4NC2** | MAQVQLVESGGGLVQAGGSLRLSCAASGLTFSRYVMGWFRQAPGKEREFVAAITWGGTPNYADSVKGRFTISRDNSKNTQYLQMNSLKPEDTAVYYCAAGLGWDSRYSQSYNYWGQGTQVTVS | TcdB-B1 |
| **4QGY** | MAQVQLVESGGGLVQAGGSLRLSCAASGFTFEDYAIGWFRQAPGKEREGVSCISNLDGSTYYPDSVKGRFTASSDKAKNMVYLQMNSLKPEDTAVYYCAAVNAQGIYCTDYIIGPYGMDYWGKGTQVTVS | type 6 secretion system TssM protein |
